# Trends in deaths following drug use in England before, during, and after the COVID-19 lockdowns

**DOI:** 10.3389/fpubh.2023.1232593

**Published:** 2023-09-29

**Authors:** Athanasios Sekeris, Thikra Algahtani, Daniyar Aldabergenov, Kirsten L. Rock, Fatima Auwal, Farah Aldewaissan, Bryn D. Williams, Nicola J. Kalk, Caroline S. Copeland

**Affiliations:** ^1^Centre for Pharmaceutical Medicine Research, Institute of Pharmaceutical Science, King’s College London, London, United Kingdom; ^2^Department of Anaesthetics, Royal Free Hospital NHS Foundation Trust, London, United Kingdom; ^3^South London and Maudsley NHS Foundation Trust, London, United Kingdom

**Keywords:** COVID-19, coronavirus, drug-related deaths, suicide, substance use disorder, opioids, methadone, antidepressants

## Abstract

**Aim:**

This research aimed to describe how the characteristics of deaths following drug use changed during the COVID-19 pandemic in England, and how this can inform future strategy to support the health and social care of people who use drugs in future emergency scenarios.

**Method:**

All deaths reported to the National Programme on Substance Abuse Deaths which occurred between January 2018 and December 2021 inclusive were extracted for analysis. Exponential smoothing models were constructed to determine any differences between forecasted vs. actual trends.

**Key results:**

Following the first lockdown period in England there were significant increases in the proportion of people who died at home beyond the 95% confidence bounds of the exponential smoothing model and concurrent decreases in the proportion of people who died in hospital. Whilst the overall proportion of deaths attributable to opioids did not significantly deviate from the forecasted trend, there were significant increases in methadone-related deaths and decreases in heroin/morphine-related death beyond the 95% confidence bounds. The proportion of deaths concluded as suicide increased, as did those implicating antidepressant use. There were no changes in the proportion of deaths following use of other drug classes, alcohol use in combination with psychoactive drugs, or on decedent demographics (gender, age, and drug user status). A small number of deaths due to drug use had COVID-19 infection itself listed as a cause of death (*n* = 23).

**Conclusion:**

For people who use drugs, the impact of the restrictions due to the COVID-19 pandemic was greater than that of infection from the virus itself. The health and social care strategy for these people needs to be pre-emptively adapted to mitigate against the specific risk factors for fatal drug overdose associated with future emergency scenarios.

## 1. Introduction

The UK government response to the COVID-19 pandemic comprised a number of strategies to mitigate the impact of the disease. These included lockdown measures, whereby socialising, travel, in-person education, and non-essential business operations were restricted, and the provision of economic support for businesses and individuals (1). The first lockdown in England was initiated on March 23rd 2020 and was gradually eased from mid-June 2020 until non-essential shops, hotels and entertainment venues were allowed to reopen on July 4th 2020 (2). A second “firebreak” lockdown was planned for November 5th – December 2nd 2020, with a third and final lockdown taking place January 6th – March 8th 2021 (2).

The impact of these measures on people who use drugs were substantial, both in terms of their drug use behaviours and how they interacted with support services. Restrictions on international travel and subsequent disruptions in the global supply chain affected the availability and purity of illicit drugs (3–9), with “stay at home” messaging increasing the visibility and detection of street drug dealers by law enforcement (7). This led to illicit drug shortages, fluctuations in their prices, and increased incidence of adulterated or substituted substances (7, 9–12). Some individuals reported changes in the types of drugs that they used (7, 9, 12–15), and also in their consumption habits due to increased psychological stress, anxiety, social isolation, and boredom due to the pandemic and the resultant restrictions (13–19). Whilst reduced access to illicit drugs will have mitigated their harms, when accessed the risk of drug overdose was likely increased as individuals were often isolated and unable to determine the strength of the substance they were now using, the dosage to administer, and the frequency that they would use (13, 14, 20, 21). To mitigate against barriers to drug treatment and support services, most patients who received daily supervised consumption of opioid agonist therapies (OAT; methadone or buprenorphine) for opioid use disorder in England were switched to one- or two-week “take home” prescriptions (7, 22), and new patients were swiftly initiated onto drug treatment programs with telehealth consultations promoted (23–25). Together these measures supported the “stay at home” message for people who use drugs – who often have multiple health co-morbidities making them particularly vulnerable to COVID-19 (26, 27) – whilst also ensuring continuity of care.

It is well established that changes in access to drugs, for example due to natural disasters (e.g., Hurricane Katrina (28)) or drug policy controls (e.g., the UK Psychoactive Substances Act 2016 (29)), impact upon the number and characteristics of deaths related to drug use (30, 31). Accordingly, an evaluation is needed to understand whether the COVID-19 pandemic and resultant social and economic restrictions had positive or negative effects on the incidence and characteristics of deaths following drug use. By doing so, it will be possible to inform the health and social care strategy needed to incorporate changes which decreased incidence of fatal drug overdose, and to mitigate against the specific risk factors for increased incidence of fatal drug overdose, upon the occurrence of future emergency scenarios.

In this study we have sought to understand the impacts of the COVID-19 pandemic on deaths related to drug use in England by analysing data held by the National Programme on Substance Abuse Deaths (NPSAD). We have presented NPSAD data where deaths occurred from the beginning of 2018 until the instigation of the first lockdown in England on March 23rd 2020 (i.e., deaths which occurred prior to any pandemic restrictions), and up until the end of 2021 (i.e., deaths which occurred peri- and post-pandemic restrictions). We aimed to examine trends in decedent demographics (age, gender, drug user status), and characteristics of deaths following drug use (location of death, cause(s) of deaths, implicated drugs, manner of death, incidence of co-morbid COVID-19 infection).

## 2. Methods

### 2.1. The National Programme on Substance Abuse Deaths

NPSAD receives reports from over 85% of English coronial jurisdictions (*n* = 70/82) for deaths related to psychoactive drug use other than nicotine or caffeine (i.e., both licensed pharmaceutical medications and illicit substances). Deaths due to alcohol use alone do not qualify for reporting to the NPSAD, but cases with combined use of alcohol and psychoactive substances do. A death is referred to a coroner if it has an unknown cause, is violent or unnatural, sudden and unexplained, occurred during an operation or before the person came out of an anaesthetic, or potentially caused by an industrial disease or poisoning (32). Coronial inquest files shared with NPSAD typically comprise the Record of Inquest (including cause(s) of death), witness statements, general practitioner (GP) records, hospital records, and post-mortem and toxicology results. Toxicology tests are usually requested in cases where drug use is suspected, at the discretion of the coroner and/or consulting pathologist.

#### 2.1.1. Ethics

The King’s College London Biomedical & Health Sciences, Dentistry, Medicine and Natural & Mathematical sciences Research Ethics Sub-Committee reconfirmed in August 2022 that NPSAD does not require ethics review as all subjects are deceased.

#### 2.1.2. Case identification

We retrospectively identified cases for analysis by extracting all deaths which occurred January 2018 – December 2021 inclusive and had been reported to NPSAD by 31st December 2022.

#### 2.1.3. Data analysis

##### 2.1.3.1. Software

Analysis was carried out using IBM^®^ SPSS^™^ Statistics for Windows version 27 and Microsoft Excel 365.

##### 2.1.3.2. Study periods

Data pertaining to deaths which occurred prior to the onset of the COVID-19 pandemic in England (January 2018 – February 2020 inclusive) were used to forecast expected trends in March 2020 – December 2021 using exponential smoothing models (*α*=0.5), with confidence bounds calculated using a 95% confidence interval. Models were constructed for each trend in the study analysis; where not graphically displayed the actual trend did not significantly deviate beyond the 95% confidence bounds of the forecasted trend. Consistent deviations outside the 95% confidence bounds were deemed significant. All percentage figures are rounded to 0 d.p.. “Lockdown” periods were defined as those where restrictions on human socialisation and movement were enforced within England (March 23rd – July 4th 2020; November 5th – December 2nd 2020; January 6th – March 8th 2021) (2).

##### 2.1.3.3. Cause of death

Circumstances that lead to death are categorised on the death certificate issued by the coroner, as follows:

Cause 1a: The immediate cause of death (and underlying if no 1b or 1c cited).
Cause 1b: Any disease/circumstances underlying Cause 1a.
Cause 1c: Any disease/circumstance underlying Cause 1b.
Cause 2: Any disease/circumstance that did not cause the death but contributed in some way.

It is not a requirement for a Cause 1b, 1c or 2 to be cited for all deaths (33). The cause of death fields were used to determine underlying cause(s) of death, and to identify implicated drugs.

## 3. Results

In this study 8,520 deaths were extracted for analysis, 54% of which (*n* = 4,625) occurred between year beginning 2018 and the day prior to the introduction of the first lockdown in the England on March 23rd 2020 as a result of the COVID-19 pandemic. The remaining 46% of deaths (*n* = 3,895) occurred from the first day of this first lockdown to year end 2021.

**Figure 1:**
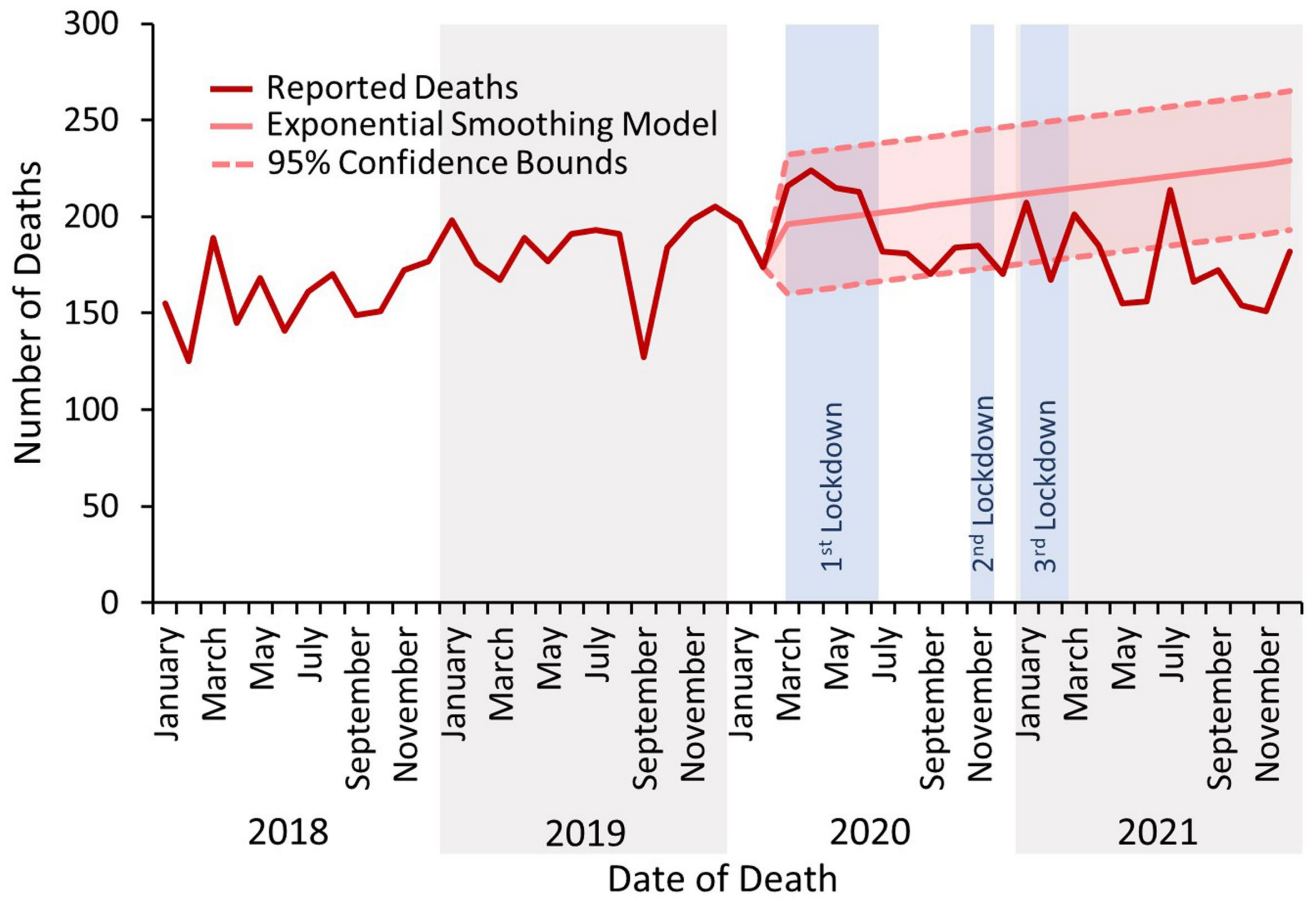
Number of deaths reported to the NPSAD from England from January 2018 to December 2021 inclusive.

**Figure 2:**
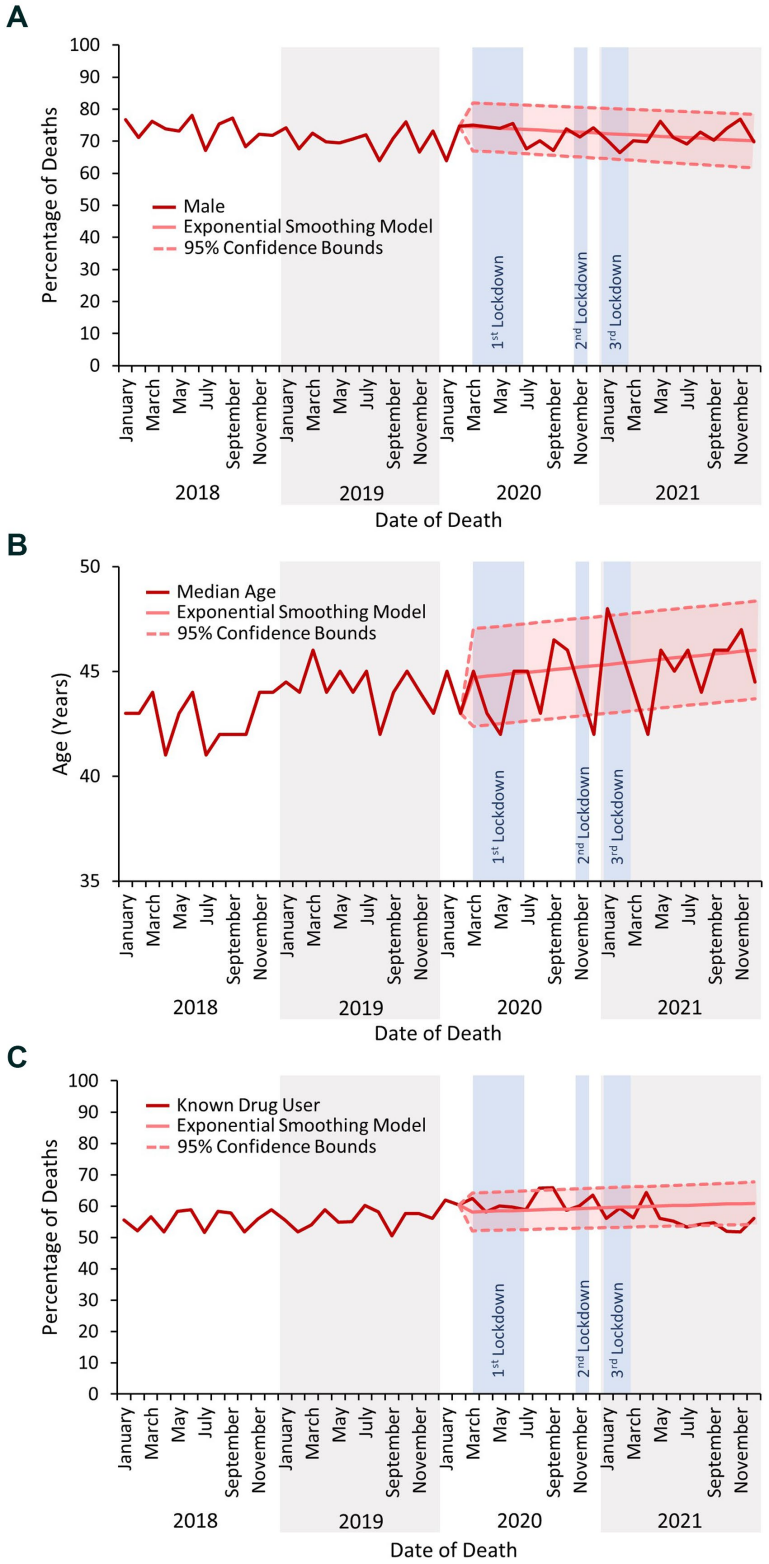
**(A)** The percentage of deaths reported to the NPSAD from England from January 2018 to December 2021 inclusive which occurred in males. **(B)** The median age of decedents reported to the NPSAD from England from January 2018 to December 2021 inclusive. **(C)** The percentage of deaths reported to the NPSAD from England from January 2018 to December 2021 inclusive which occurred in known drug users.

The number of deaths related to drug use reported to the NPSAD following the first COVID-19 lockdown did not continue to increase as per the previous and forecasted trends (Figure 1). Rather, after the mean number of reported deaths increased from 183 per month in 2019 to 193 per month in 2020, they decreased to 176 per month in 2021. Decedents continued to be predominantly male (Figure 2A), with median age at death generally increasing in line with the previous and forecasted trends (Figure 2B), although there were incidences where this fell below the 95% confidence bounds of the exponential smoothing model. The proportion of decedents who were known to have used drugs did not significantly change during any of the lockdown periods, with the proportion of known drug users remaining consistent as opposed to the slight increase predicted by the exponential smoothing model (mean proportion of known drug users 2018 – February 2020: 56%; March 2020 – December 2021: 58%; Figure 2C).

### 3.1. Place of death

**Figure 3:**
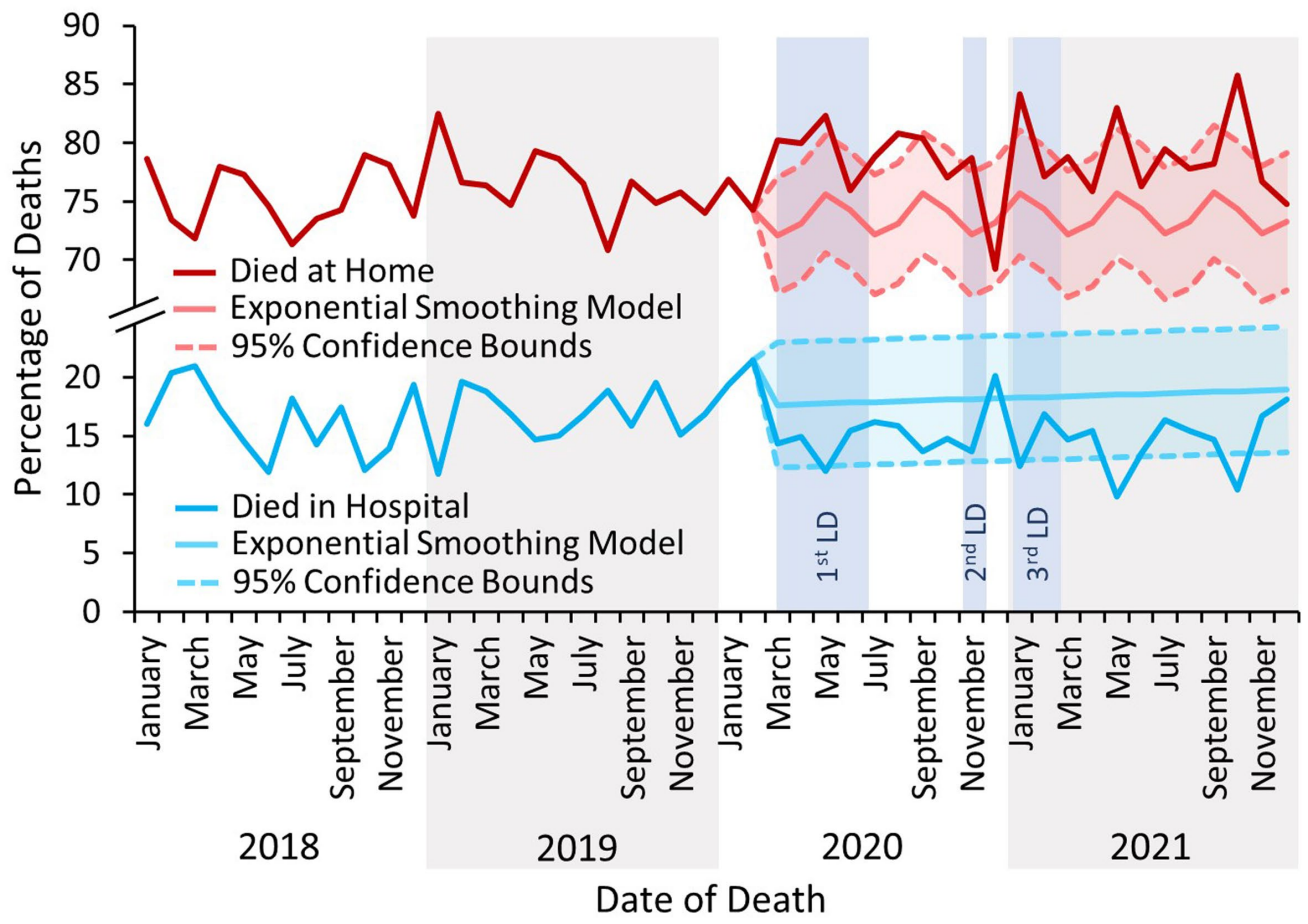
The percentage of deaths reported to the NPSAD from England from January 2018 to December 2021 inclusive which occurred at the decedents’ homes or in hospital. LD – Lockdown. There is considerable seasonality evident in the forecasted trend for deaths in the home, with peaks around January, May, and September of each year. Whilst these months correspond to the end of the major school holiday periods in England (Christmas, Easter, and Summer) and warrant further investigation, this is outside the scope of the present study.

The proportion of people who died at home increased beyond the bounds of the 95% confidence interval of the exponential smoothing model based on previous trends following the first COVID-19 lockdown and remained elevated throughout 2020–21 (Figure 3). Contrastingly, the proportion of people who died in hospital decreased below the lower bound of the forecasted rate, which also persisted throughout 2021.

### 3.2. Manner and cause of death

**Figure 4:**
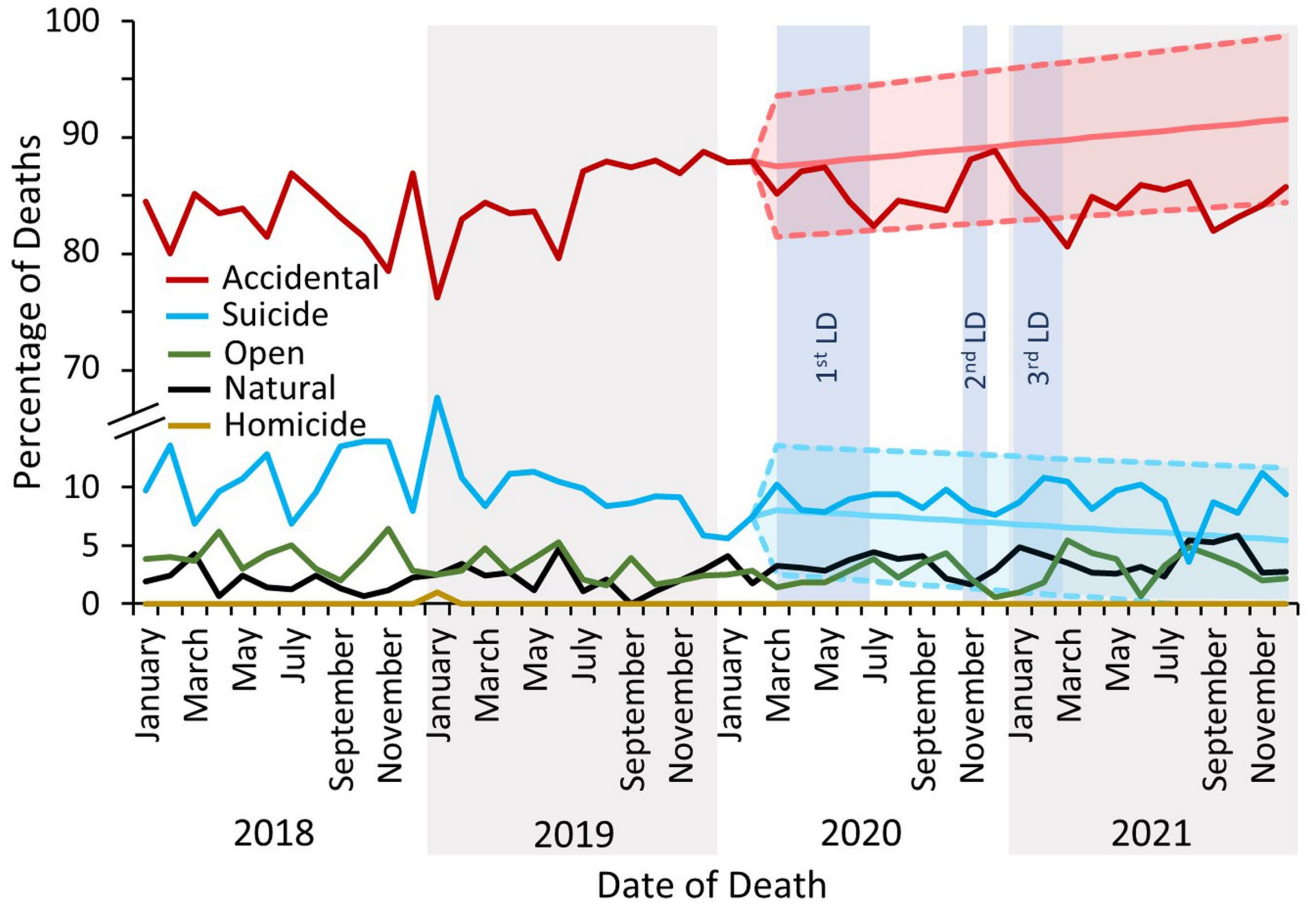
The percentage of deaths reported to the NPSAD from England from January 2018 to December 2021 inclusive by inquest conclusion. The exponential smoothing model and 95% confidence bounds for the accidental death trend is indicated by the light red shaded area, and for the suicide trend by the light turquoise area. Exponential smoothing models and 95% confidence bounds were calculated for open, natural and homicide trends, but were not significantly different from the actual proportion of reported deaths and are not displayed. LD, lockdown.

Deaths due to drug use were predominantly concluded at inquest as accidental in nature throughout the study period (Figure 4). However, the proportion of deaths concluded as suicide following the first COVID-19 lockdown was elevated above the forecasted rate – although remained within the upper bound of the 95% confidence interval – and corresponds with the concomitant decrease in the proportion of accidental death conclusions (Figure 4). There were no significant changes in the proportion of inquests concluded as natural, open (i.e., where intent could not be determined) or homicidal in nature (Figure 4).

**Figure 5:**
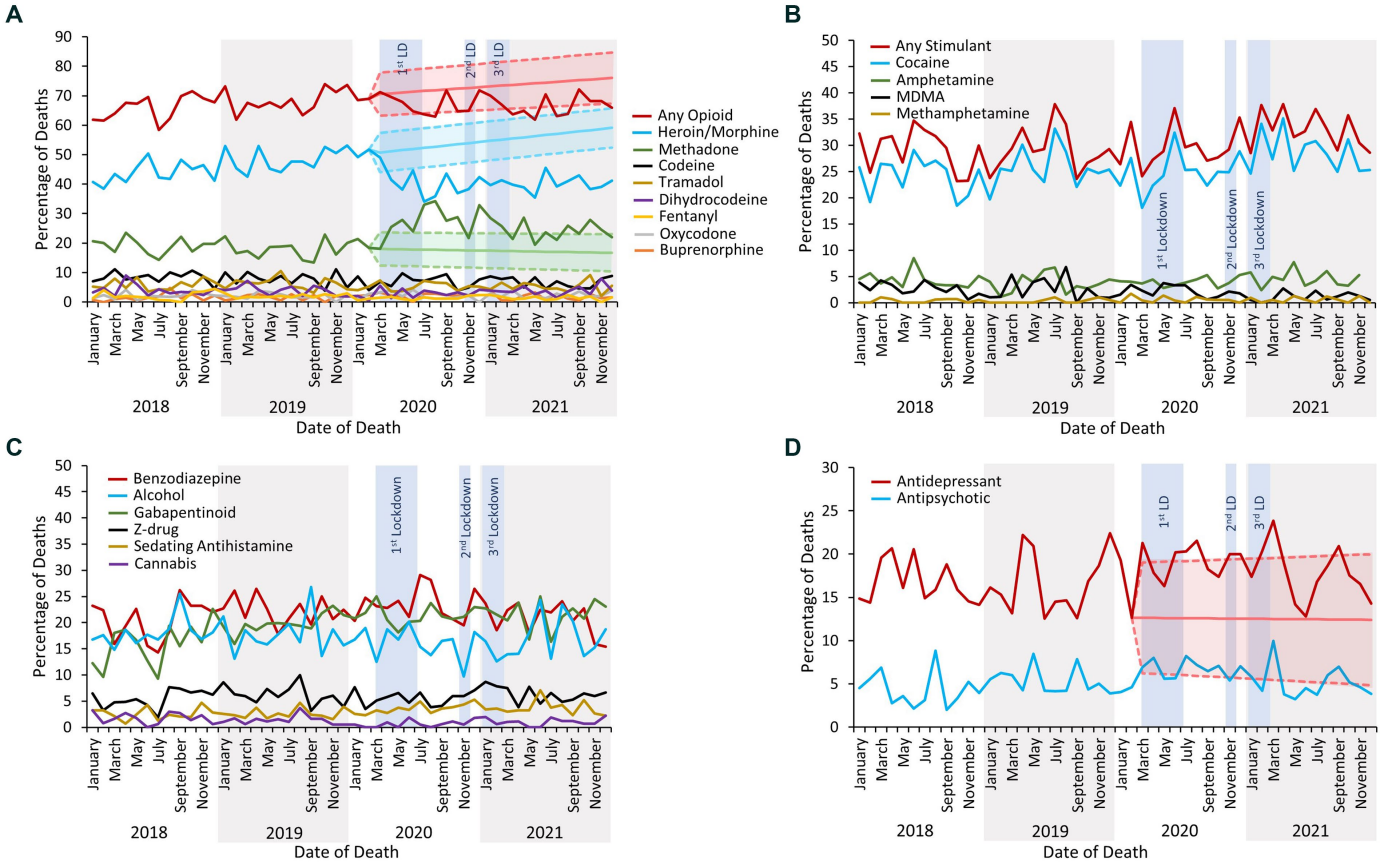
**(A)** The percentage of deaths reported to the NPSAD from England from January 2018 to December 2021 inclusive due to use of any opioid and by individual opioid. The exponential smoothing model and 95% confidence bounds for deaths due to use of any opioid is indicated by the light red shaded area, for deaths due to use of heroin/morphine by the light turquoise shaded area, and for deaths due to use of methadone by the light green shaded area. Exponential smoothing models and 95% confidence bounds were calculated for the remaining individual opioids but were not significantly different from the actual proportion of reported deaths and are not displayed. **(B)** The percentage of deaths reported to the NPSAD from England from January 2018 to December 2021 inclusive due to use of any stimulant and by individual stimulant. Exponential smoothing models and 95% confidence bounds were calculated for each trend but were not significantly different from the actual proportions of reported deaths and are not displayed. **(C)** The percentage of deaths reported to the NPSAD from England from January 2018 to December 2021 inclusive due to use of other sedative drugs and alcohol. Exponential smoothing models and 95% confidence bounds were calculated for each trend but were not significantly different from the actual proportions of reported deaths and are not displayed. **(D)** The percentage of deaths reported to the NPSAD from England from January 2018 to December 2021 inclusive due to use of antidepressants and/or antipsychotics. The exponential smoothing model and 95% confidence bounds for deaths due to use of antidepressants is indicated by the light red shaded area. An exponential smoothing model and 95% confidence bounds were calculated for the antipsychotic-related death trend but were not significantly different from the actual proportions of reported deaths and are not displayed. LD, lockdown.

Opioids were the class of drug most commonly implicated in causing death throughout the study period. Following the first COVID-19 lockdown the overall proportion of deaths due to opioid use slightly decreased (2019 average of 125 deaths per month, 2021 average of 117 deaths per month,) instead of increase as forecasted. However, sub-analysis by opioid type revealed that whilst the proportion of deaths due to heroin/morphine consumption decreased, there was a concurrent increase in deaths due to methadone use (note: as heroin is rapidly metabolised to 6-monoacetylmorphine and morphine (34), it cannot be determined whether heroin or morphine was administered if heroin-specific markers are not tested for – as such, heroin and morphine are combined in the NPSAD database). There were no significant changes in the proportions of deaths due to use of other major opioid drugs (Figure 5A).

Stimulants were the second largest class of drug implicated in causing death, with the majority of stimulant-related deaths related to cocaine use (Figure 5B). There were no significant changes in the proportions of deaths due to stimulant drug use following the first COVID-19 lockdown, either when considered as a group or by individual substance (Figure 5B). Similarly, there were no significant changes in the proportion of deaths due to the use of other drugs which can induce sedation, or deaths where alcohol was implicated in combination with psychoactive drug use (Figure 5C).

**Figure 6:**
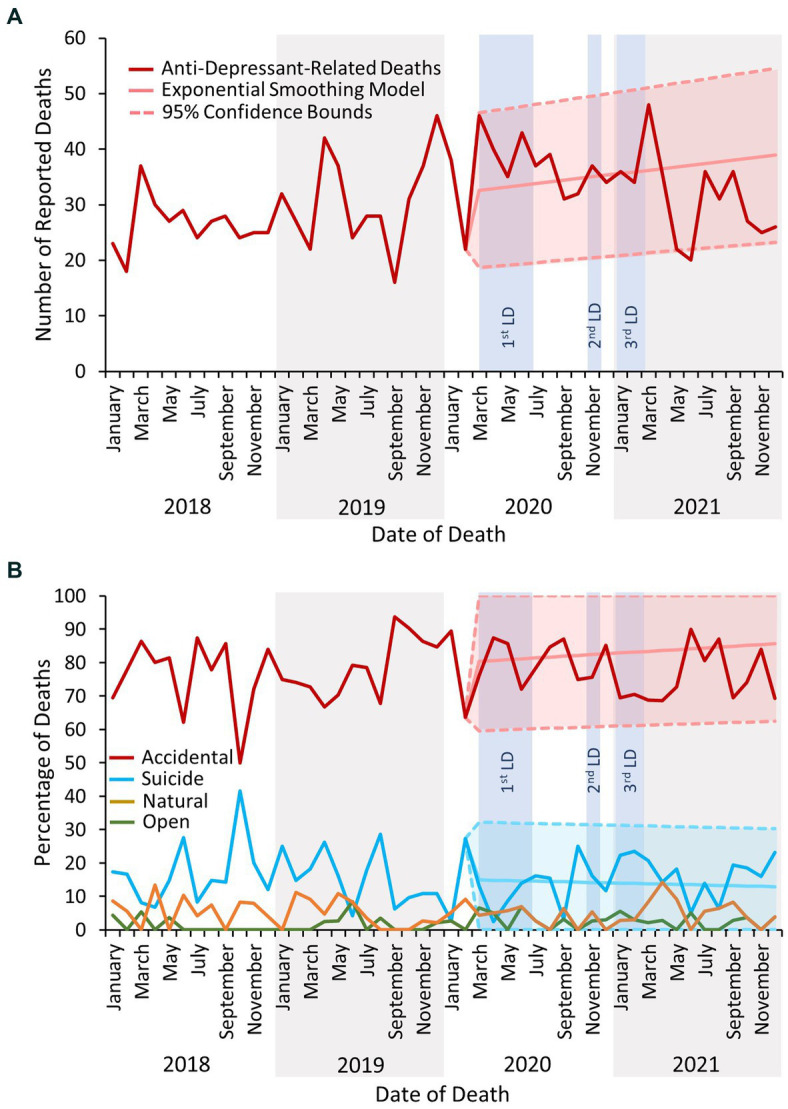
**(A)** The number of deaths reported to the NPSAD from England from January 2018 to December 2021 inclusive due to antidepressant use. **(B)** The percentage of deaths due to antidepressant use reported to the NPSAD from England from January 2018 to December 2021 inclusive by inquest conclusion. The exponential smoothing model and 95% confidence bounds for the accidental death trend is indicated by the light red shaded area, and for the suicide trend by the light turquoise area. Exponential smoothing models and 95% confidence bounds were calculated for open and natural trends but were not significantly different from the actual proportion of reported deaths and are not displayed. No antidepressant-related deaths were reported to the NPSAD which were deemed homicidal in nature. LD, lockdown.

Deaths due to antidepressant drug use were significantly elevated above the forecasted 95% confidence bounds multiple times during and immediately following the lockdown periods (Figure 5D). Indeed, the raw number of reported deaths increased (January 2018 – February 2019 mean deaths per month: 29; March 2020 – March 2021 mean: 38; Figure 6A) although the proportion of these deaths concluded as suicide did not increase (Figure 6B). There were no significant changes in the proportions of deaths due to the use of antipsychotic drugs (Figure 5D).

COVID-19 was listed as a cause of death in 24 cases (<1% of deaths included in the study post-March 23rd 2020). In 13 of these cases COVID-19 was cited as an underlying cause of death (*n* = 3 sole underlying cause of death; *n* = 10 underlying cause of death in combination with acute drug toxicity). In the remaining 11 cases COVID-19 was cited as a contributary factor to death occurring (i.e., as a Cause 2): in 10 cases it was deemed to have contributed to death occurring due to the respiratory infection caused by the disease, and in 1 case due to exacerbation of the deceased’s anxiety and depression due to the pandemic.

## 4. Discussion

Regarding deaths due to drug use, the COVID-19 pandemic was associated with an increase in the proportion of people who died at home vs. in hospital, and with changes to the characteristics of fatal opioid overdose. The proportion of deaths concluded as suicide slightly increased, as did the proportion of deaths following antidepressant use. There were no changes in the proportion of deaths following the use of other drug classes, or the gender, age, or drug user status of decedents. A very small number of deaths had COVID-19 infection itself listed as a cause of death.

### 4.1. Remote monitoring strategies are needed to mitigate risk of lone drug use

The increase in the proportion of people who died at home is understandable given the “stay at home” messaging prevalent throughout the COVID-19 lockdowns (2), a phenomenon also observed in opioid overdoses the US (35) and in deaths in the wider general population in England and Wales (36). A pre-pandemic estimate that 61% of opioid use takes place in the presence of others (37) likely decreased during and following lockdown periods (38, 39). There is recognised increased risk of death associated with lone drug use (40, 41); however, this was the precise condition enforced by the pandemic restrictions. Measures that facilitate remote monitoring, such as “spotting” via video call (42), or via wearable devices that can detect overdose pathophysiology and alert emergency services (43, 44), may mitigate the risks of lone drug use.

### 4.2. A case of could not attend or would not attend hospital?

Hospital attendance by adult patients in England during the first lockdown decreased by ~42%, with a 31% reduction in attendances needing immediate attention to save life, such as drug overdoses (45). Lower rates of hospital attendance due to drug overdose could be due to increased incidence of lone drug use – whereby there is nobody present to call for help (40, 41) – compounded with increasing emergency responder times in England over the course of the pandemic (46). Furthermore, people suffering with health conditions as a result of chronic IV drug use, such as sepsis or endocarditis (26, 27), may have been hesitant to attend hospital for fear of contracting COVID-19 (45) and died without help at home. Indeed, fear of contracting COVID-19 amongst the wider population likely contributed to more people who in a non-pandemic year may have typically died in hospital, dying at home (36).

### 4.3. People with dual diagnoses need concurrent support

Whilst the pandemic had significant impacts on mental health globally (47, 48), people who use drugs faced additional challenges including anxieties over accessing drugs, treatment provision, and support services (13–19). Between 40 and 70% of adults with a substance use disorder have at least one mental health disorder (49, 50), which likely increased in co-incidence during the pandemic (15, 17, 19). Indeed, this study found an increase in deaths by suicide and due to antidepressant use following the onset of the pandemic, and further evidences the need for Drug & Alcohol and Mental Health services to deliver concurrent care for people with such a “dual diagnosis” (51). The use of telehealth in the provision of these services during the COVID-19 pandemic generally proved successful (52–55) and is therefore an appropriate model for service delivery during a future emergency scenario.

### 4.4. Fatal opioid overdose characteristics changed despite no change in overall prevalence

A previous study examining only the first 3 months of the first lockdown found a significant increase in methadone-related death, with the majority increase due to deaths in people not prescribed methadone (22). The extended timeframe in this study demonstrates that this significant increase persisted into 2021, with a concurrent significant decrease in heroin/morphine-related death. Most patients in England receiving daily doses of methadone via supervised consumption were switched to one- or two-week “take-home” prescriptions at the beginning of the pandemic to mitigate against pharmacy closures and risk of COVID-19 infection in a vulnerable patient group, and support the “stay at home” guidance (22). Whilst higher volumes of drug dispensing in the community correlates with their increased misuse (56–61), there are many factors that need considering when interpreting this trend. These include, for example, reduced hospital attendance over the pandemic (36, 45, 62), although reduced access to treatment for opioid dependence does not appear to have been a factor in England (22). It is possible that people prescribed methadone were taking more than the intended dose due to lack of supervision, although incidence of methadone-related death in people prescribed methadone did not increase (22). Alternatively, people prescribed methadone could have been diverting their dose, contributing to the replacement of heroin in the drug market with methadone, as evidenced by heroin/morphine-related deaths reducing and people who use drugs reporting that heroin was less available, more expensive, and of lower quality in the UK during the pandemic (7, 63).

### 4.5. Closing of recreational spaces did not change fatalities associated with “recreational” drug use

Deaths related to “party drugs” – stimulant drugs, such as cocaine, amphetamine, and MDMA (64) – did not decrease during the pandemic despite the closing of recreational spaces and prohibition of socialising (1). However, the motivations for stimulant drug use are not always purely recreational (64), as they also offer an escape from the stressors of life (65–69), or from unbearable situations such as homelessness, abuse, or incarceration (70–75). The persistence of stimulant-related death during the pandemic may therefore comprise a reduction in recreational “party drug” use and an increase in dependent drug use (e.g., cocaine in its “crack” form (76)).

It may be surprising that deaths following alcohol use did not change following the first lockdown, as it is well documented that alcohol use increased for many people (77–80). However, NPSAD reporting criteria is dependent upon psychoactive drug use, so any alcohol-related deaths are actually combined drug- and alcohol-related. Furthermore, most alcohol-related deaths occur due to established alcohol-related liver disease as opposed to acute alcohol poisoning (81).

### 4.6. Strengths and limitations

The strengths of this study lie in the granularity of the NPSAD data – the provision of full toxicology reports enables the drugs involved in each death to be determined, including those with ambiguous causes of death such as “multi-drug toxicity”. However, the NPSAD does not receive reports from every coronial jurisdiction in England, although the high compliance rate (over 85%) supports that the study findings can be considered representative for the whole cohort. To this end, not all deaths are referred to a coroner, and only a proportion of those referred undergo toxicology testing. Therefore, even with a 100% coronial reporting rate, data would still only reflect the proportion of deaths referred and subjected to toxicology testing. Furthermore, whilst most toxicology labs screen for common illicit drugs and licensed medications, there are variations between labs as to which substances can be detected. It is also possible that reporting jurisdictions have not concluded all inquests from deaths which occurred during the study period. Therefore, more deaths from the study period may still be reported, although as this study was performed with all deaths reported by December 31st 2022 – a year on from the end of the study period – the vast majority of 2021 deaths from reporting jurisdictions will have been received. Accordingly, the exponential smoothing model predictions made were based upon the data readily available at time of writing, and may be subject to change as and when further deaths from the study period are reported.

## 5. Conclusion

The impact of the circumstances posed by the COVID-19 pandemic was greater than that of infection from the virus itself for people who use drugs. Whilst this study suggests that deaths related to drug use in England plateaued – albeit at an all-time high (82) – following the onset of the pandemic, the factors underlying this trend evolved, and so must the health and social care strategy to prevent fatal drug overdose. The findings of this study indicate that advances in digital healthcare, such as the remote monitoring of people who use drugs to mitigate the risks of lone drug use, and telemedicine consultations to enable patient engagement with physical health, substance use, and mental health services, currently represent the best adaptive responses for future emergency scenarios.

## Data availability statement

The data analyzed in this study is subject to the following licenses/restrictions: the dataset will be available upon reasonable request from the corresponding author. Requests to access these datasets should be directed to CC caroline.copeland@kcl.ac.uk.

## Ethics statement

The requirement of ethical approval was waived by The King’s College London Biomedical & Health Sciences, Dentistry, Medicine and Natural & Mathematical sciences Research Ethics Sub-Committee for the studies involving humans because the Data Privacy Act and GDPR do not apply to personal data once a person is deceased and therefore does not require ethical review (see methods Section 2.1.1). The studies were conducted in accordance with the local legislation and institutional requirements. Written informed consent for participation was not required from the participants or the participants’ legal guardians/next of kin because The Data Privacy Act and GDPR do not apply to personal data once a person is deceased and therefore does not require ethical review (see methods Section 2.1.1).

## Author contributions

AS collected the data, performed the analysis, wrote the first draft, and revised the manuscript. TA, DA, KR, FatA, and FarA collected the data and revised the manuscript. BW, NK, and CC conceived the idea and revised the manuscript. All authors contributed to the article and approved the submitted version.

## Conflict of interest

The authors declare that the research was conducted in the absence of any commercial or financial relationships that could be construed as a potential conflict of interest.

## Publisher’s note

All claims expressed in this article are solely those of the authors and do not necessarily represent those of their affiliated organizations, or those of the publisher, the editors and the reviewers. Any product that may be evaluated in this article, or claim that may be made by its manufacturer, is not guaranteed or endorsed by the publisher.
